# The role of PaCO₂ in high-altitude pre-acclimatization

**DOI:** 10.1093/nsr/nwaf243

**Published:** 2025-06-28

**Authors:** Dawei Shi, Jing Chen, Meitong Li, Lingling Zhu, Xunming Ji

**Affiliations:** School of Automation, Beijing Institute of Technology, China; School of Automation, Beijing Institute of Technology, China; School of Automation, Beijing Institute of Technology, China; Institute of Military Cognition and Brain Sciences, Academy of Military Medical Sciences, China; Department of Neurosurgery, Xuanwu Hospital, Capital Medical University, China

We thank Prof. Burtscher and colleagues for their thoughtful letter regarding our review article entitled ‘Closing the loop: autonomous intelligent control for hypoxia pre-acclimatization and high-altitude health management’ [1]. We acknowledge that the interaction between peripheral and central chemoreceptor responses, along with their regulation of arterial CO₂ partial pressure (PaCO_2_), indeed offers deeper insights into the progression of acclimatization. In this context, measuring PaCO₂ can provide critical physiological information beyond what can be inferred from peripheral oxygen saturation (SpO_2_) alone.

Inspired by Prof. Burtscher and colleagues’ perspective, we reviewed the recent progress of PaCO_2_ measurement technologies to evaluate the applicability in closed-loop pre-acclimatization, in which transcutaneous technology appears to be a promising option. Transcutaneous CO₂ sensors estimate arterial tension by measuring CO₂ diffusing out through the skin surface [2]. Traditional devices use a heated Severinghaus electrode placed on the ear lobe or chest to ‘arterialize’ local blood flow and facilitate CO₂ diffusion [3]. Commercial systems such as the food and drug administration (FDA)-cleared SenTec Digital Monitor and Radiometer TCM series confirm the clinical maturity. However, these systems typically exhibit gradual measurement drift that needs to be frequently calibrated. A risk of local skin irritation or burns caused by the heated sensor also reduces user compliance.

To overcome these issues, newer optical sensing approaches are emerging to offer faster and more stable CO₂ measurements [3]. These optical sensors are able to track physiological CO₂ changes on the timescale of minutes [2,3], with fewer calibrations and negligible sensitivity to humidity or O₂ interference [4]. Despite this, such systems remain at a prototype or engineering-sample stage. In a recent review article [3], 13 studies were included to discuss optical transcutaneous PaCO_2_ measurement. Only 7 involved human subjects and none received FDA clearance, which indicates that the application of optical sensors in PaCO_2_ monitoring still requires substantial translational research.

Apart from transcutaneous technology, measuring end-tidal CO₂ (EtCO₂) is a common surrogate for estimating PaCO_2_. It involves collecting exhaled gas by using a face mask and analysing it through the infrared analysis of the gas at end-exhalation. In healthy individuals, EtCO₂ usually trails PaCO₂ by 2–5 mmHg; however, this gap can be enlarged due to hypoxia-induced ventilation–perfusion mismatch or shallow breathing, which causes degraded measurement inaccuracy [5]. Although EtCO₂ monitoring is now widely integrated into intensive care unit ventilators [6,7], hypoxicators used for pre-acclimatization lack this capability, which may influence the development of closed-loop pre-acclimatization that incorporates PaCO_2_ monitoring.

In conclusion, incorporating reliable PaCO₂ monitoring into the closed-loop pre-acclimatization controller appears both technically feasible and clinically advantageous for indicating a more accurate acclimatization status prior to high-altitude exposure. However, the development of user-friendly commercial devices with accurate and real-time PaCO_2_-monitoring capabilities still remains an important step toward enabling practical high-altitude pre-acclimatization. Accordingly, the exploration of autonomous intelligent control approaches to integrate such critical physiological information from SpO_2_, PaCO_2_ holds great potential, which can benefit from continued advances in measurement technologies.
